# Bipolar cemented hip hemiarthroplasty in patients with femoral neck fracture who are on hemodialysis is associated with risk of stem migration

**DOI:** 10.3109/17453670902875237

**Published:** 2009-04-01

**Authors:** Jan Blacha, Robert Kolodziej, Marek Karwanski

**Affiliations:** ^1^Orthopedics and Traumatology Department, Medical University of LublinUniversity Hospital no. 4, LublinPoland; ^2^Institute of Economic Informatics, Warsaw University of Life SciencesWarsawPoland

## Abstract

**Background and purpose** Femoral neck fractures are considerably more common in patients on hemodialysis than in the general population. We determined the outcome of bipolar hemiarthroplasty for hip fracture in patients with long-term hemodialysis and compared it with that of a matched-paired group of patients with intact renal function.

**Methods** We analyzed 26 bipolar hemiarthroplasties in 23 hemodialysed patients with a mean age of 56 (41–78) years who were followed for mean 3.6 (1–8) years. These cases were matched for age, sex, and BMI with 26 patients with femoral neck fractures and normal renal function. The mean follow-up time in the control group was 7 (5–10) years. As primary surgery for their femoral neck fracture, all patients had a bipolar hemiarthroplasty with a 28-mm metal-polyethylene internal articulation and a cemented stem.

**Results** The mortality rate was 21% in the hemodialysed group and 4% in the control group (p = 0.005). The bipolar head migrated in 1 patient in the hemodialysed group but in none of the patients in the case-matched group. In the hemodialysed group, 8 stems migrated and 3 of these were revised, whereas in the control group 3 stems migrated and 2 were revised. The stem migration in the hemodialysed group was not preceded by the development of osteolysis or radiolucent lines at the bone-cement interface. The cumulative survival for prosthesis migration as endpoint was 44% at 5 years in the hemodialysed group and 96% in the control group (p = 0.03).

**Interpretation** The main mode of failure of cemented bipolar hemiarthroplasties in hemodialysed patients is stem migration, due to failure of the bone-cement interface.

## Introduction

In patients on hemodialysis the incidence of femoral neck fracture is rated to be 7 per 1,000 patient-years in males and 17 per 1,000 in females, which is approximately 4 times higher than that observed in the general population (Alem et al. 2000, Kaneko et al. 2006). It has also been reported that the cumulative incidence of hip arthroplasty in dialysis patients is 35 episodes per 10,000 person-years as compared to 5 in the general population (Abbot et al. 2003).

The surgical treatment alternatives for femoral neck fracture in patients who are on chronic hemodialysis are osteosynthesis and arthroplasty. Several authors have recommended arthroplasty because of a high risk of failure after internal fixation (Hardy et al. 1994, Kalra et al. 2006, Karaeminogullari et al. 2007).

There have been several studies reporting the outcome of hemiarthroplasty or total hip arthroplasty in patients with chronic renal failure, but with contrasting results (Schaab et al. 1990, Hardy et al. 1994, Lieberman et al. 1995, Bednarek-Skublewska et al. 2003, Sakabe et al. 2006, Karaeminogullari et al. 2007). Most of these studies involved a small number of subjects and did not analyze the modes of failure of the implant.

We analyzed the modes of failure, prevalence of migration, and rates of revision of bipolar hemiarthroplasty in patients on long-term hemodialysis and compared the results to those from a match-paired group of patients with no kidney disease.

## Patients and methods

### Demographics

Between 1996 and 2005, we performed 26 consecutive cemented bipolar hemiarthroplasties in 23 patients with intracapsular femoral neck fracture who were on hemodialysis for chronic renal failure. 12 of the fractures were not associated with trauma.

The causes of renal failure were chronic glomerulonephritis (11), pyelonephritis (3), diabetes mellitus (3), systemic lupus erythematosus (3), polycystic kidney disease (2), and renal tuberculosis (1). 5 patients had had 1 failed renal transplant attempt before the study. None of the patients had a functioning renal transplant at the time of fracture or during follow-up. 6 patients were chronically ill with multiple medical problems, but preoperative ambulation was preserved in all patients. The average age at surgery was 56 (41–78) years and the average follow-up time was 3.6 (1–8) years. The mean duration of hemodialysis prior to bipolar arthroplasty was 11 (2–26) years.

The 23 patients on dialysis (with 26 bipolar arthroplasties) were match-paired with 26 patients with 26 bipolar arthroplasties that were performed after traumatic femoral neck fracture. Minimum follow-up time in the control group was 5 years. Patients were match-paired for sex, age, and BMI. The match-paired control patients were selected at random from a consecutive series of 115 patients with femoral neck fracture treated with bipolar hemiarthroplasty at our institution from 1993 through 2000. The renal function of these patients was intact. We found factors that predisposed 3 patients in the control group to fracture (steroid therapy due to asthma in 1 patient, and 2 other patients were heavy smokers).

The average age in the control group at surgery was 60 (41–77) years and the mean follow-up was 7 (5–10) years. The details of hemodialysis, the status of any renal transplant, and all data concerning the medical condition of the patient related to dialysis were gathered retrospectively from the hospital records. All other clinical and radiological data concerning the patients were collected prospectively (Table 1).

**Table 1.** Demographics of patients on hemodialysis who underwent bipolar hemiarthroplasty for femoral neck fracture, and of matched controls

	Hemodialysis	Control
No. of patients (hips)	23 (26)	26 (26)
Males/females (hips)	12/11 (13/13)	10/16 (10/16)
Age mean (range)	56 (41–78)	60 (41–77)
BMI mean (SD)	24 (3.7)	27 (4.1)
Mean follow-up (range) in months	43 (1–94)	88 (60–115)
No. of deaths: patients (hips)	5 (7)	1 (1)
Time between fracture and death in months, mean (range)	37 (1–65)	87
Mean duration of dialysis (range) in months	121 (27–312)	NA ^a^

^a^ Not applicable.

All patients underwent surgery at our institution using a posterolateral approach; this was performed by 8 experienced orthopedic surgeons. Each patient received the same type of prosthesis: a cemented stem (Ultima; Johnson and Johnson) and a bipolar head (Monk type) with polyethylene 28-mm metal inner articulation (Johnson and Johnson). The median external size of the heads used was 50 (44–56) mm. The same cementing technique was used in both groups, including broaching of the femoral canal, insertion of a distal restrictor, and injection of cement (Surgical Simplex or Antibiotic Simplex P; Stryker, Howmedica, Osteonics).

The University Hospital in Lublin is a certified hemodialysis referral center. Each patient underwent hemodialysis on the day before surgery, without heparinization. All patients were managed pre- and postoperatively by a nephrologist team to ensure the best medical condition before and after surgery. Patients on hemodialysis did not receive subcutaneous LMWH prophylactically. Patients in the control group received subcutaneous LMWH for 14–30 days. Prophylactic antibiotics (second generation of cephalosporin) were given intravenously on the day before surgery and treatment was continued for 3–5 days postoperatively in all patients. The suction drain was removed 24–72 hours after surgery. Patients in the hemodialysed group were requested to maintain 50–75% weight bearing for 6 weeks with the aid of crutches, at which time they were allowed to bear full weight. The patients in the control group were allowed to bear full weight as tolerated.

All patients were evaluated preoperatively and postoperatively at 3–6 months, 12 months, and annually thereafter. Pain and function were recorded at each follow-up visit using the Harris hip score.

### Radiographic evaluation

After operation, anteroposterior radiographs of both hips were routinely taken annually or whenever clinical symptoms indicated. For the patients who died, the last available radiograph was used in the analysis. Radiographs were digitized and migration was measured using an image analysis program (Scion Image for Windows; Scion Corp., Fredrick, MD). The known diameter of the bipolar head was used to determine the magnification on radiographs. The position of the femoral component was assessed by comparing the positions of reference points on the prosthesis and on the tip of the greater trochanter. Subsidence was recorded as being present when the femoral component migrated more than 3 mm or varus/valgus displacement of more than 5 degrees was noted.

Bipolar head migration was evaluated by drawing a horizontal line through the inferior aspect of the bilateral teardrops and a vertical line through the medial aspect of the teardrop. The medial or proximal migration was determined by measuring the perpendicular distance from the lines to the top and medial aspects of the bipolar head. Head migration was recorded when the distance measured was more than 3 mm and there was no radiolucent line representing cartilage in acetabulum. All radiographs were evaluated by both authors. Endpoints considered in this study included radiographic migration of the stem or bipolar head and revision for any reason.

### Statistics

Mean, standard deviation, median, and range were calculated for all continuous variables. The generalized linear mixed model (GLMM) procedure was used to fit statistical models for dependent samples. It was used to compare proportions of events occurring in different groups. We used odds ratio measures obtained from GLMM with logit link function and normal assumption about error. To fulfill the condition of sample dependency, estimation was done using compound-symmetry block dispersion matrix. Matching pairs were included directly into models as strata.

The Cox proportional-hazards model and Kaplan-Meier test were used to analyze survival data. The model was fitted using robust sandwich estimator due to repeated measures. The stratified analysis was used to perform correction for the match-paired strategy. Cumulative survival rates for the two groups were compared using the log-rank test. Regression analysis was performed to analyze the effect of sex, age, BMI, and duration of hemodialysis on migration of the prosthesis. A p-value of less than 0.05 was considered statistically significant. Calculations were carried out using the GLIMMIX and TPHREG procedures from the SAS package (version 9.1) and using Statistica (version 6).

## Results

All of the patients in both groups were satisfied with the results of bipolar hemiarthroplasty and were functioning well up until migration of the prosthesis. The main success was pain relief. We observed that the patients from the hemodialysed group became slightly more debilitated during the follow-up period, in a progressive manner. At the time of the final follow-up, however, 16 of 26 hips in the 23 hemodialysed patients functioned well without any prosthesis migration, and with a Harris hip score (HHS) of more than 80. The mean HHS was 90 (82–100). The hemodialysed patients who had migration of any prosthesis component had lower Harris hip scores: mean 61 (32–73) (p = 0.001).

We did not compare clinical function between the groups because of substantial differences in the general health status and activity levels of the patients.

### Complications

In the hemodialysed patients, there was 1 intraoperative undisplaced fracture of the greater trochanter, postoperative hematoma in 5 hips, and 1 superficial infection with prolonged antibiotic administration for 9 days. There were 2 hematomas in the control group. The difference in complication rates was not statistically significant (p = 0.2).

None of the patients had deep vein thrombosis, thromboembolism, or iatrogenic nerve injury. There were no dislocations in any patients. There were no periprosthetic fractures during follow-up in either of the groups.

5 of the 23 patients in the hemodialysed group died (7 hips). 1 patient died of cardiopulmonary arrest 16 days after surgery, and 4 patients died between 1.5 and 5 years after surgery (6 hips). None of the deaths were related to hip surgery. In the control group, 1 patient died 7 years after surgery. The difference in mortality rate between the groups was statistically significant (p = 0.005).

### Radiographic analysis (Table 2).

*Acetabular side.* In the hemodialysed group, 1 patient had medial (8 mm) and proximal (13 mm) migration of the bipolar head at 3 years after operation. The prosthesis was revised with an anti-protrusio cage, with good outcome. None of the patients in the control group had migration of the bipolar head.

**Table 2.** Details of migrated bipolar prostheses in patients from both groups

Case no.	Group	Sex	Age at operation (years)	Duration of dialysis (months)	BMI	Head size/neck length	Time to migration (months)	Time to revision (months)	Migrated element
1	dialysis	M	46	133	23	46/short	23	28	stem
2	dialysis	M	49	170	23	48/medium	9	not done	stem
3	dialysis	F	41	30	35	46/medium	58	63	stem
4	dialysis	M	44	204	28	52/medium	1	1	stem
5	dialysis	M	58	298	22	52/medium	18	not done	stem
6	dialysis	M	59	130	23	50/short	12	not done	stem
7	dialysis	F	41	29	20	4/short	13	not done	stem
8	dialysis	F	50	48	24	48/short	37	53	head
9	dialysis	M	54	75	26	48/medium	62	not done	stem
10	trauma	M	41	na	26	50/short	92	103	stem
11	trauma	F	64	na	25	48/medium	97	105	stem
12	trauma	F	65	na	27	44/short	46	not done	stem

*Stem side.* We noticed migration of 8 stems in the hemodialysed group. The mean distal migration was 14 mm (6–23) mm; 1 stem had additional valgus displacement of 11 degrees. Only 3 of 8 stems that migrated were revised, because of contraindications for surgery. In addition, 5 patients had minor clinical symptoms. The stem migration was not preceded by the development of osteolysis or radiolucent lines at the bone-cement interface. Long cemented stems (205–240 mm) were implanted in all revised cases. The acetabulum cartilage was intact in all revised cases and a new bipolar head was implanted. There were no macroscopic signs of wear of the internal polyethylene surface in the bipolar heads that were removed.

In the control group, 3 patients had radiographic signs of stem migration. The mean stem subsidence was 13 (8–21) mm and valgus displacement of 9 and 10 degrees was noticed in 2 of the 3 migrated stems. In all these cases, migration followed osteolysis around the stem. 2 of the stems were successfully revised using long cemented stems (220 and 240 mm). All removed bipolar heads had substantial macroscopic signs of polyethylene wear.

*Prosthesis survival.* In the hemodialysed patients, the cumulative survival with migration as an endpoint was 44% (95% CI: 14–90) at 5 years. It was 96% (95% CI: 47–100) in the control group at 5 years and 77% (95% CI: 42–98) at 9 years (p = 0.03, Figure).

**Figure:**
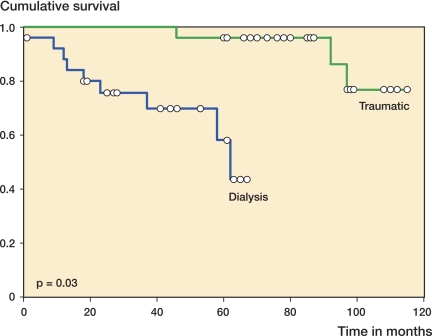
Cumulative survivorship curve estimated for bipolar hemiarthroplasty in hemodialysed and trauma patients with migration for any reason as an endpoint. Differences were significant (p = 0.03).

We did not calculate the cumulative survival rate with revision as endpoint because many of the hemodialysed patients with prosthesis migration had serious contraindications to revision operation. Regression analysis of age, sex, BMI, and duration of dialysis in relation to migration did not reveal any significant associations.

## Discussion

Our patients had a long history of hemodialysis. The end-stage of renal disease, chronic hemodialysis, and transplantation failure may cause multiorgan insufficiency, metabolic imbalance, and a high risk of postoperative complications and mortality. Despite the high risk of complications, operative treatment of femoral neck fracture has been recommended in this group of patients (Tzamaloukas et al. 1990). Pathology associated with hemodialysis may lead to early loosening of the implant because of poor bone quality or migration of amyloid deposits into the bone-implant interface (Crawford and Athanasou 1998). The literature on this subject is not extensive. It is also unclear how renal insufficiency affects the acetabular cartilage and its ability to articulate with a bipolar head.

Only 1 of the 23 patients on hemodialysis died during the first postoperative year. Higher mortality rates (10–100%) have been reported (Schaab et al. 1990, Klein et al. 1998, Sunday et al. 2002, Kalra et al. 2006, Sakabe et al. 2006, Karaeminogullari et al. 2007). We must stress that all of our patients were treated postoperatively by an expert team of nephrologists. Our results are in accordance with those presented by Nagoya et al. (2005).

The prefracture ambulation status is a significant predictor of life expectancy after femoral neck fracture in hemodialysed patients (Sakabe et al. 2006). All in from our series were active and lived independently.

Reduced immune reactivity in patients on long-term hemodialysis may increase the risk of infection (Naito et al. 1994, Lieberman et al. 1995, Sakalkale et al. 1999). None of our hemodialysed patients had a deep infection diagnosed. Low infection rates in such patients were also reported by Karaeminogullari et al. (2007) and Nagoya et al. (2005).

The literature contains conflicting results regarding clinical and radiographic outcome of hip arthroplasty in patients on hemodialysis. Lieberman et al. (1995) reported poor results in 13 of 16 patients, Naito et al. (1994) presented poor results in 6 of 15 patients, whereas Gualteri et al. (1995) showed 6 good or excellent results in a series of 8 patients. Hardy et al. (1994) obtained excellent long-term results in 11 of 13 cases of hemiarthroplasty. Sakalkale et al. (1999) reported good-to-excellent clinical results for 11 of 15 hips in 12 patients despite the fact that 7 patients had early complications.

The clinical outcome of total hip arthroplasty (THA) in a group of patients with renal transplant was reported to be better than in patients on hemodialysis (Gualtieri et al. 1995, Lieberman et al. 1995). However, Shrader et al. (2006) reported a higher incidence of complications and revisions in renal transplant patients than in hemodialysed patients with THAs.

In our series of patients, the main mode of prosthesis failure in both groups was stem migration. In the control group, we observed development of radiolucencies and osteolysis around the stem before migration occurred. In the revised cases, we observed wear of internal metal-polyethylene articulation, severe osteolysis at the cement-bone interface of the femur, and well-preserved cartilage in the acetabulum. In the hemodialysed patients, migration of the stem occurred without radiographic evidence of osteolysis. This indicates that subsidence of the stem was due to unsatisfactory support to the bone and failure of the bone-cement interface. Pathology associated with long-term hemodialysis may lead to poor bone quality, which may fail to support the implant.

Naito et al. (1994) reported loosening of cemented prostheses in 5 of 15 hips after an average of 5 years follow-up. Toomey and Toomey (1998) reported migration of 8/15 cemented stems with a mean time to failure of 8 years. Karaeminogullari et al. (2007) reported a cumulative survival of 63% at 32 months, of 8 unipolar and bipolar hip arthroplasties that were performed for femoral neck fracture. Nagoya et al. (2005) reported no loosening of 11 uncemented stems and suggested that the use of uncemented implants with extensively coated stems may prevent loosening of the femoral components. On the other hand, Gualtieri et al. (1995) recommended the use of cemented stems in hemodialysis patients because of poor bone quality. Sakalkale et al. (1999) reported no revision of the cemented or cementless femoral stem in hemodialysed patients who had undergone total hip arthroplasty for osteoarthritis or osteonecrosis. It should be noted that the quality of bone in these patients may be different from that in hemodialysed patients with femoral neck fracture.

It has been reported that both the patient’s age and the age at onset of hemodialysis may have a considerable influence on the functional outcome in patients with femoral neck fracture who are also undergoing hemodialysis (Sakabe et al. 2006). We could not, however, find any relationship between migration of the prosthesis and the age of the patient, or between migration and the duration of hemodialysis.

Based on the results of our study, it appears that cemented bipolar hemiarthroplasty in patients on hemodialysis (performed by an orthopedic surgeon with postoperative management including a nephrologist and a physiotherapist) helps to reduce postoperative complications and mortality. Most of the patients had a good or excellent clinical outcome. However, the cumulative survival with prosthesis migration as an endpoint was substantially lower than in patients with normal renal function, due to stem migration not associated with osteolysis. Adequate stability of the stem is a crucial consideration for satisfactory long-term results of bipolar hemiarthroplasty in patients on hemodialysis. Thus, improvements in stem fixation are needed.
